# Template-free synthesis of salmon pink tube-shaped structure carbon nitride with enhanced visible light photocatalytic activity†

**DOI:** 10.1039/c8ra09950b

**Published:** 2019-01-25

**Authors:** Youzhi Cao, Xinbo Jing, Yajuan Chen, Wenjie Kang, Shufen Wang, Wei Wang

**Affiliations:** School of Chemistry and Chemical Engineering, Key Laboratory for Green Processing of Chemical Engineering of Xinjiang Bingtuan, Shihezi University Shihezi 832003 China wangwei_group@sina.com; College of Sciences, Shihezi University Shihezi 832003 China

## Abstract

Designing a highly active and stable photocatalyst to directly solve environmental pollution is desirable for solar energy conversion. Herein, an effective strategy, hydrothermal-calcination, for synthesizing extremely active carbon nitride (salmon pink) from a low-cost precursor melamine, is reported. The salmon pink carbon nitride with tube-shaped structure significantly enhanced response to visible light, improved efficiency of charge separation and remarkably enhanced efficiency of methyl orange (MO) degradation than bulk g-C_3_N_4_ (light orange). The M-10-200-24-600 composite possessed the most wonderful ability towards MO degradation irradiated by visible light, which could achieve a highest degradation efficiency of 84% within 120 min. Our findings may provide a promising and facile approach to highly efficient photocatalysis for solar-energy conversion.

## Abstract 2

Designing a highly active and stable photocatalyst to directly solve environmental pollution is desirable for solar energy conversion.

## Graphical abstract

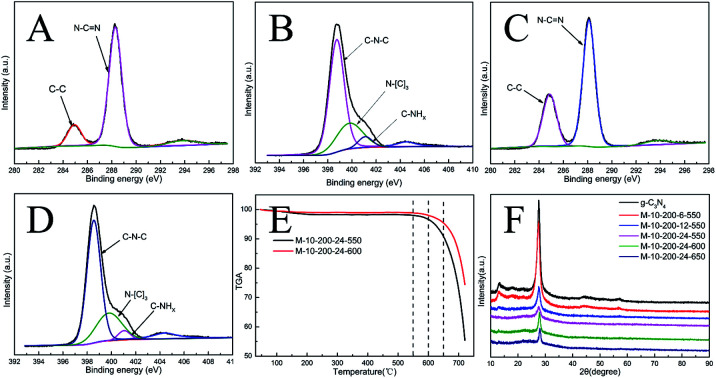

## Introduction

The increase in environmental pollution and the depletion of fossil resources has resulted in an ever-growing demand for green and unlimited solar energy, and this has triggered an enormous amount of research on the development of highly efficient photocatalysts under the current global background of the increase in environmental pollution and the depletion of fossil resources. Over the past years we have witnessed the rapid development of visible-light driven photocatalysis which is generally regarded as one of the promising technologies for large-scale solar energy capture and the delivery of alternative energy carriers to fossil fuels. It also has enormous potential in environment cleaning and for energy production.^1–3^

Developing active and stable visible-light responsive photocatalyst is crucial for successful implementation of these applications. Among the various visible-light responsive materials, metal-free g-C_3_N_4_ has drawn immense concern for its suitable bandgap of 2.7 eV, low cost, ease of preparation, good stability, environmental friendly feature and directly absorb visible light to drive chemical reactions, thus leading to multifunctional application for photocatalytic degradation of pollutants, photocatalytic hydrogen generation, carbon dioxide reduction as well as supercapacitors.^4–9^

Nevertheless, the shortcomings of low visible light utilization efficiency and fast recombination of photogenerated electron–hole pairs in bulk g-C_3_N_4_ still limit the further improvement in its photocatalytic activity. In order to improve the photocatalytic performance of g-C_3_N_4_, enormous efforts have been devoted to improving the photocatalytic activity of g-C_3_N_4_, such as nano/mesoporous structures design, doping with metal or non-metal elements, surface modification and heterostructured nanocomposites fabrication, and so forth.^10–14^ As we all know, the g-C_3_N_4_ nanosheets always exhibit more excellent performance than bulk g-C_3_N_4_ (similar to graphene and graphite). However, the nanonization process may cause enlarged bandgap due to the quantum size effect.^15–18^ To maximize the photocatalytic conversion efficiency, carbon nitride with diverse structures have been prepared and utilized, such as rod,^19–24^ sheets^25–27^ and spheres,^28^ in which tube-shaped structure has been proved to be an appealing and effective strategy.^29–31^ Two methods are mostly employed in preparing tubular carbon nitride. The first one involves template-free synthesis of tubular carbon nitride.^32–35^ The second method is to use assisted templates such as hard templates and soft templates, but requires the removal of templates. Thus, developing a facile strategy to synthesize tubular carbon nitride is of vital importance to further develop its catalytic mechanism and potential applications. Zou and coworkers successfully synthesized a carbon nitride intercalation compound by heating the melamine with a low melting point eutectic mixed salts (LiCl·H_2_O–KCl–NaCl) under air and ambient pressure. Interestingly, g-C_3_N_4_ nanotubes was produced. The resultant g-C_3_N_4_ nanotubes is very stable and active for solar H_2_ production.^36^

**Scheme 1:**
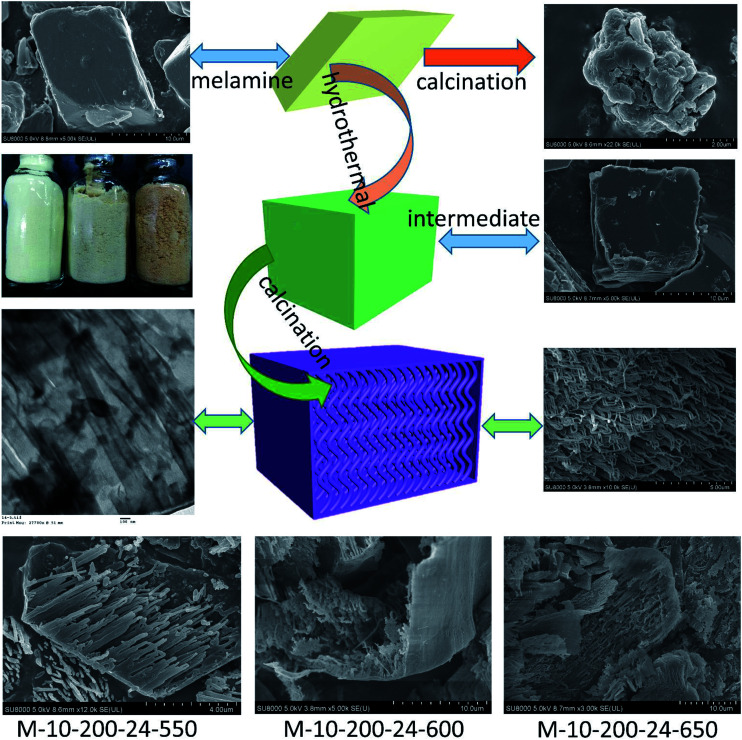
The process for preparation of the tube-shaped structure carbon nitride through successively hydrothermal treatment and calcination.

Herein, we report a facile and effective approach for the fabrication of tube-shaped architecture carbon nitride, which was used as photocatalyst for methyl orange (MO) degradation. As illustrated in Scheme 1, the tube-shaped structure carbon nitride was synthesized through hydrothermal treatment and calcination and showed excellent photocatalytic performance.

## Experimental

### Materials

Melamine (C_2_H_4_N_4_, CP), methyl orange (MO, AR), and other chemicals involved were purchased from Sinopharm Chemical Reagent Co., Ltd. (China) and used directly for experimental without further purifications. All aqueous solutions throughout were prepared with the deionized water.

### Catalysts preparation

10 g melamine was mixed with 50 mL deionized water, then the suspension was transferred into a 100 mL Teflon-lined stainless autoclave and heated at 200 °C (160, 180) for 24 (6, 12)h in an oven. After rapid cooling to room temperature, the product was collected by evaporation in 60 °C and denoted as M-10-200-24. The intermediate was put into an alumina crucible with a cover and then heated to 550 (600, 650)°C with a heating rate of 2.5 °C min^−1^ for 4 hours under the Ar atmosphere, followed by rapid cooling to room temperature to yield the M-10-200-24-550 product. Respectively, For comparison, bulk g-C_3_N_4_ was also prepared with melamine placed in an alumina crucible and calcined at 550 °C for 4 h in Ar atmosphere at a heating rate of 2.5 °C min^−1^, and then the product was ground to a homogeneous powder.

### Catalysts characterization

X-ray diffraction (XRD) of the samples was carried out on a Bruker AXS D8 X-ray diffractometer with a Cu-Kα X-ray source operating at 40 kV and 100 mA. The morphologies of the samples were observed using a scanning electron microscope (SEM, JEOL JSM-6490LV) and a transmission electron microscope (TEM, FEI Tecnai G2). X-ray photoelectron spectroscopic (XPS) measurements were made on an Escalab 250Xi system. UV-vis diffuse reflection spectra of the as-prepared products were carried out using an Evolution 220 UV-vis spectrophotometer (Thermo Fisher) from 200 to 800 nm. Fourier transform infrared (FT-IR) spectra of the samples were measured by a Nicolet 5700 Fourier transform infrared spectrometer. The photoluminescence spectra (PL) of the samples were obtained using a fluorescence spectrometer (Hitachi F-4500) at 293 K.

### Photocatalytic experiments

The activity of the photocatalysts was evaluated *via* the photocatalytic degradation of MO under visible light irradiation. In a typical experiment, 0.05 g photocatalyst was suspended in 100 mL 10 mg L^−1^ MO solution. The suspension was first dispersed by sonication for 15 min and stirred for 15 min in the dark to obtain adsorption–desorption equilibrium between the MO and the photocatalyst. The suspension was then stirred and irradiated under a 300 W Xe lamp (*λ* > 400 nm). During the irradiation process, about 3 mL of suspension was taken from the reaction cell every 20 min and centrifuged to remove the photocatalyst. The absorbance of the MO solution in degradation was detected on TU-1900 UV-vis spectrophotometer.

## Results and discussion

Melamine has three amino groups and weakly alkaline, which can react with acid to produce melamine salt. In Fig. S1,† the melamine before and after the hydrothermal treatment was added to the hydrochloric acid solution, we can find a milky white suspension in the present of melamine without hydrothermal process. While no aforementioned phenomenon can be observed after using melamine with hydrothermal treatment. The phenomenon indicates the amino was break in the hydrothermal process, it can be proved by FT-IR (Fig. S2†). The hydrothermal process makes a significant influence on the morphology of carbon nitride. In Scheme 1, the SEM images reveal a bulk structure of g-C_3_N_4_. After hydrothermal treatment, the crystalline form of melamine has been altered and a new intermediate formed, which is consistent with the result of XRD patterns (Fig. S3†). Following thermal polymerization, a typical structure of carbon nitride is obtained. However, carbon nitride was synthesized in different polymerization temperature, most of them show a typical tube-shaped structure. Besides, a shell was found in the M-10-200-550, M-10-200-600, and M-10-200-650 samples, a possible formation mechanism is that the shell of carbon nitride was formed by direct polycondensation using the outer wall of the intermediate. While the internal tube-shaped structure is maybe due to the shrink of the interior space of intermediate with the NH_3_ release and calcination temperature elevating. After directly heating melamine to 550 °C in Ar atmosphere, the color of carbon nitride turns into yellow, which is consistent with the enlarged bandgap (Fig. 2C). After heating intermediate to 550, 600, 650 °C in Ar atmosphere, the color of the resulting carbon nitride gradually turns into light yellow, light orange and salmon pink caused by the defect structure in tube-shaped carbon nitride, resulting from the decomposition of some amine groups and triazine ring, which can be confirmed by the following characterizations. The darker color of M-10-200-24-650 indicates a better absorption of visible light, which is consistent with the result of UV-vis absorption spectra (Fig. 2B).^15^

**Fig. 1:**
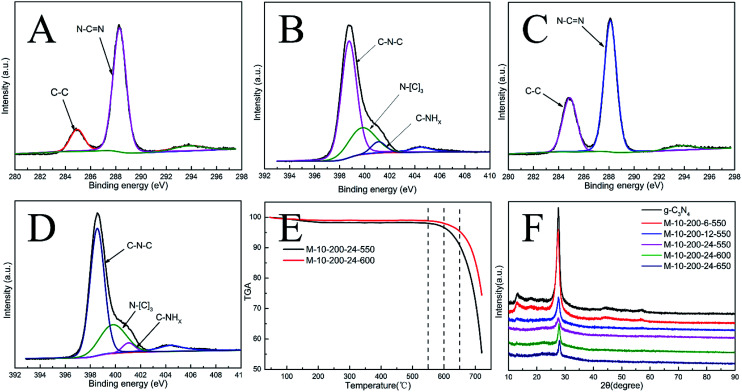
(A) High-resolution XPS of C 1s and (B) N 1s for g-C_3_N_4_. (C) High-resolution XPS of C 1 s and (D) N 1s for M-10-200-24-600. (E) TGA for M-10-200-24-550 and M-10-200-24-600. (F) XRD pattern of the samples.

**Fig. 2:**
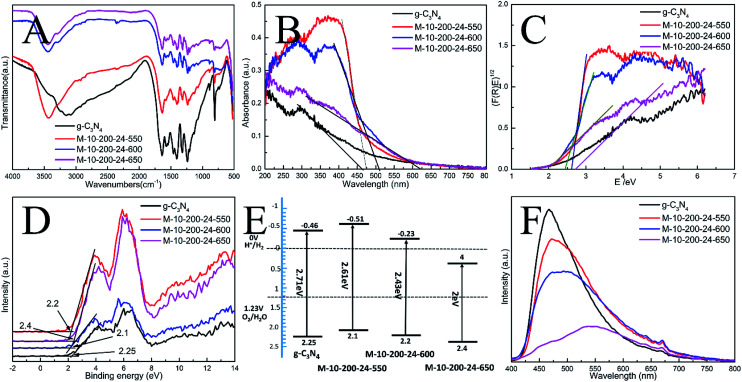
(A) FT-IR spectra, (B) UV-vis DRS, (C) the plots of (F(R)E)^1/2^, (D) XPS valence band spectra, (E) schematic diagrams of the energy band structure and (F) PL spectra of the as-prepared catalysts.

X-ray photoelectron spectroscopy (XPS) is used to further probe the chemical states of the g-C_3_N_4_ and M-10-200-24-600 samples. From the C 1s spectrum (Fig. S4A†) for the bulk g-C_3_N_4_, the mainly carbon species centering at 284.6 eV and 288.2 eV are assigned to graphitic carbon (C–C) and sp^2^-hybridized carbon in the N-containing aromatic ring (N–C=N), respectively. Compared with bulk g-C_3_N_4_, the proportion of C–C increase and C–N=C decrease in M-10-200-24-600 (Fig. S4C†), indicating the introduction of the defects and the cleavage of carbon nitride. For the N 1s spectrum in Fig. S4B and D†, a small peak located at 404.2 eV is attributed to the positive charge localization in the heterocycles; the bigger one starting from 396.4 eV to 402.7 eV can be deconvoluted into three peaks, the peaks with binding energy of 398.8, 400.2, and 401.4 eV correspond to two-coordinated N(C–N–C), three-coordinated N(N–(C)_3_), and N–H structure, respectively. As the deconvolution results listed in Table S1,† we found that the intensity of binding energy at 398.8 eV increased and the intensity of those at 400.1 and 401.2 eV decreased, which indicates the cleavage of N–(C)_3_ and the destroy of C–NH.

**Fig. 3:**
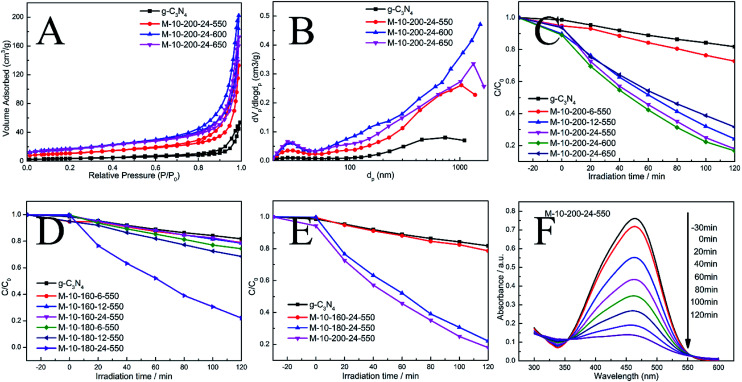
(A) N_2_ adsorption–desorption isotherms and (B) corresponding pore size distribution curves of the samples. (C)–(E) Photocatalytic performances for the degradation of MO over samples under visible light irradiation (*λ* > 400 nm). (F) The absorption spectral changes of MO by M-10-200-24-550.

The surface atomic C/N ratios of the g-C_3_N_4_, M-10-200-24-550, M-10-200-24-600 and M-10-200-24-650 samples are 0.70, 0.68, 0.88 and0.67, respectively. Clearly, with the polymerization temperature rising from 550 to 600 °C in Ar atmosphere, the C/N molar ratio increased from 0.68 to 0.88 for the decomposition of partial nitrogenous species, which leads to the unique rich carbon structure. However, when the polymerization temperature increased to 650 °C, the C/N molar ratio decreased due to the volatilization of carbon species, detailed data is shown in Table S1.† Thermogravimetric analysis (TGA) (Fig. 1E) indicates that the main structure of M-10-200-24-550 and M-10-200-24-600 still being kept well at 600 in N_2_ atmosphere.

Representative X-ray diffraction (XRD) patterns of the bulk g-C_3_N_4_ and the products obtained are shown in Fig. 1D. All the samples show two typical diffraction peaks. Bulk g-C_3_N_4_ gives two typical diffraction peaks at around 13.1° and 27.2° as reported previously, which are respectively due to the in-plane structural packing motif (100) and periodic stacking (002) of layers along the *c*-axis.^15^ With the thermal treatment of the intermediate in an Ar atmosphere, these two sharp peaks significantly decrease and only a weak peak appears near 27.2°, suggesting that the order degree of in-plane structural unit decreases resulting from losing some bridging HN(C) or NH_2_ units. A possible reason for the low crystallinity of tube-shaped structure carbon nitride is most likely resulted from the incomplete polymerization. This result confirmed the XPS analysis. In addition, the planar atomic structure of carbon nitride has been destroyed by high temperature thermal condensation method.

The interior molecular structures of products were further characterized by Fourier Transform Infrared (FT-IR) spectrometer, which was sensitive to the local structure of materials. The FT-IR spectra of g-C_3_N_4_ and series of products in Fig. 2A suggest a stable conjugated structure of the CN skeleton. A pronounced absorption at 1200–1600 cm^−1^ is attributed to the stretching vibration of CN heterocyclic; in particular, the broad band located at 2900–3300 cm^−1^ is related to residual N–H components and O–H bands, associated with uncondensed amino groups and surface-adsorbed H_2_O molecules. Whereas, the sharp peak at 802 cm^−1^ is always recognized as the typical breathing vibration of tris–triazine units, which signifies a complete skeleton g-structure of g-C_3_N_4_. In comparison with bulk g-C_3_N_4_, the vibrations of prepared samples are less intensive, suggesting the destruction of the skeleton of carbon nitride, which is consistent with the results of XPS and XRD. However, the FT-IR peaks of the samples were weaker than those of bulk g-C_3_N_4_, which might be due to the lower density of the samples or possibly a lower degree of structural order.

In order to understand the photoinduced carrier separation mechanism, the energy band structure including band gaps, conduction band (CB) and valence band (VB) positions of bulk g-C_3_N_4_, M-10-200-24-550, M-10-200-24-600 and M-10-200-24-650 samples are determined. The optical property and band structure of the samples are characterized by VB-XPS plots and UV-vis diffuse reflectance spectra (DRS) (Fig. 2B–D). After heating melamine at 550 and 600 °C in an Ar atmosphere, the light absorption of the obtained M-10-200-24-550 and M-10-200-24-600 show an obvious red shift compared to that of bulk g-C_3_N_4_. The bandgap of M-10-200-24-550 and M-10-200-24-600 obtained from Kubelka–Munk function is about 2.61 and 2.43 eV, which is much lower than that of bulk g-C_3_N_4_ (2.71 eV) inspiringly, after heating melamine at 650 °C in an Ar atmosphere, an obvious tail absorption in longer wavelength (450–750 nm) was observed in UV-vis DRS of the obtained M-10-200-24-650. The M-10-200-24-650 possessed a narrower band gap (2 eV) than other samples, allowing increased utilization of the solar spectrum and the potential to generate more photogenerated electron–hole pairs under visible light. The enhanced light absorption of tube-shaped structure carbon nitride, compared with bulk g-C_3_N_4_, is most likely due to the incomplete polymerization and the rich carbon structure.

XPS valence band spectra were analyzed to investigate the band edges of the bulk g-C_3_N_4_ and prepared samples. As shown in Fig. 2D, the valence band (VB) values of the bulk g-C_3_N_4_, M-10-200-24-550, M-10-200-24-600 and M-10-200-24-600 samples were, respectively, approximately 2.25, 2.2, 2.1 and 2.4 eV. Based on the band gaps obtained from the Tauc plots, the conduction band (CB) values of the bulk g-C_3_N_4_ and prepared samples were calculated to be −0.46, −0.51, −0.23 and 0.4 eV, respectively, compared with the vacuum energy level. The energy band positions of the bulk g-C_3_N_4_ and the tube-shaped structure carbon nitride samples are schematically shown in Fig. 2E. It is obvious that the shift in the CB and VB of the M-10-200-24-600 and M-10-200-24-550 led to a larger activity for photocatalytic redox reactions (Fig. 2C and E).

The efficiency of electron–hole pairs trapping, migration, and transfer are the key parameters in determining the photocatalytic performance, and were next invested by room temperature photoluminescence (PL) spectra. Fig. 2F shows the PL emission spectra evolution with the increase in heating power. The peak shapes of the PL spectra are similar, and the emission peaks of the bulk g-C_3_N_4_ and the products prepared at 550, 600 and 650 °C power are located at 467, 472, 484, and 551 nm, respectively. The center of the PL is constantly red-shifted with the increase in heating temperature, which could be attributed to the degree of polycondensation and the increase in the disordered structure of the carbon nitrides. However, the emission intensity reduces in the following order: bulk g-C_3_N_4_ > M-10-200-24-550 > M-10-200-24-600 > M-10-200-24-650. This trend indicates that the density of the charge carrier traps in M-10-200-24-650 should be the lowest, which is beneficial to photocatalytic reactions and consistent with the result of UV-vis DRS. Thus, the fast charge recombination, a historical intrinsic drawback of g-C_3_N_4_ photocatalyst, can be largely overcome by combination of hydrothermal treatment and high temperature thermalcondensation method.

The N_2_ adsorption–desorption isotherms and pore-size distribution curves of the prepared samples were evaluated using the BET isotherms (Fig. 3A and B). Each sample shows a typical type IV isotherm, indicating the presence of mesopores. The pore size distribution of the samples further confirms the formation of mesopores. Mesopores and macropores can be observed in the all sample. There is a significant increase in *S*_BET_ from 13.7 m^2^ g^−1^ for g-CN to 39.3, 60.9 and 59 m^2^ g^−1^ for M-10-200-24-550, M-10-200-24-600 and M-10-200-24-650. Similarly, the pore volume increases from 0.07 cm^3^ g^−1^ for bulk g-C_3_N_4_ to 0.20, 0.30 and 0.26 cm^3^ g^−1^ for M-10-200-24-550, M-10-200-24-600 and M-10-200-24-650 (Table S2†), respectively. It is obvious that M-10-200-24-600 has the largest surface area and pore volume among all the samples.

Through the hydrothermal treatment and tuning copolymerization temperature, a number of rich carbon structures can be incorporated into carbon nitride networks, resulting in a broadened range of visible light response and more efficient charge carrier separation, and therefore, better photocatalytic activity. The photocatalytic performance of the bulk g-C_3_N_4_ and the prepared samples were investigated by evaluating photocatalytic degradation methylic orange with visible light (*λ* > 400 nm). The photocatalytic activity of the bulk g-C_3_N_4_ and the prepared samples is shown in Fig. 3C–E. We studied the influence of the hydrothermal temperature, hydrothermal time and polycondensation temperature in the prepared samples on the photocatalytic activity under the same conditions. Reference experiments showed that no reactions occur when the system was illuminated in the absence of catalyst or in the presence of catalyst without illumination (Fig. S4†).

As shown in Fig. 3C–E, it is obvious that hydrothermal temperature and hydrothermal time have a significant impact to the performance of carbon nitride, which is mainly due to the conversion efficiency from melamine to intermediate. XRD date can explain the phenomenon. Meanwhile, the polymerization temperature also influences the performance of carbon nitride. We found M-10-200-24-550 and M-10-200-24-600 have excellent properties with the degradation of 84% in 2 hours, which is almost 4 times higher than that of the bulk g-C_3_N_4_ under the same conditions, among this two samples, M-10-200-24-600 shows the best activity. However, the poor photocatalytic activity of M-10-200-24-650 may be due to the triazing ring broken in high temperature of 650 °C and the reduction of active sites, which is consistent with the result of XRD, FT-IR and XPS. Based on the above analysis, an obvious increase in photocatalytic activity is caused by an enlarged surface area, shortened electron–hole transfer distance, and increased visible-light harvesting.^37^

As known, the semiconductor photocatalysis process is composed of the semiconductor photoexcitation, the separation and transfer of photoinduced charge carriers, and the subsequent photocatalytic reaction. The photoexcitation primarily rests with the bandgap structure of the semiconductor. The carbon-rich structure has successfully induced intrinsic electronic and band structure modulation of the carbon nitride. The CBM and VBM potentials for the M-10-200-24-600 were located at −0.23 eV and 2.2 eV, respectively. Thus, the M-10-200-24-600 with a narrow bandgap would harvest more visible-light photons and produce more photogenerated carriers than the bulk g-C_3_N_4_.

After photoexcitation, the photocatalytic efficiency strongly depends on the competition between photogenerated carrier recombination and photogenerated carrier separation. It is a common fact that the photogenerated holes is main effect and could be effectively consumed by the MO using the tube-shaped structure carbon nitride. Separation efficiency of the photogenerated electron–hole pairs would be promoted in this system. The creation of a tube-shaped structure can provide more surface active sites for adsorption and decomposition of reactants. Similar to the nanorod structure of the g-C_3_N_4_ photocatalyst,^19,23^ the tube-shaped structure suppress formation of recombination centers and electron–hole recombination probability, as proved by the PL analysis.

## Conclusions

In conclusion, we have generated the salmon pink carbon nitride with tube-shaped structure through a hydrothermal-calcination strategy, which presents the high-efficiency visible light photocatalytic behavior for methyl orange (MO) degradation with a rate more than that of other 2D g-C_3_N_4_ nanostructures. The detailed characterization exhibited that this synthetic method not only improved the response to visible light of the sample but also enhanced the photogenerated charge carrier mobility, as well as increased the surface area. The excellent photocatalytic performance indicates that the material may be used as an efficient and sustainable photocatalyst for alleviating global environmental and energy issues. This strategy offers new opportunities for developing the photocatalytic performance of carbon nitride.

## Conflicts of interest

There are no conflicts to declare.

## Supplementary Material

RA-009-C8RA09950B-s001
