# Supplementary material for: Template-free synthesis of salmon pink tube-shaped structure carbon nitride with enhanced visible light photocatalytic activity
Source: RSC Adv. 2019 Jan 25;9(6):3396–402. doi: 10.1039/c8ra09950b (PMC9060291; doi:10.1039/c8ra09950b)
Supplement: RA-009-C8RA09950B-s001 [file RA-009-C8RA09950B-s001.pdf]

Electronic Supplementary Material (ESI) for RSC Advances.
This journal is © The Royal Society of Chemistry 2019

Electronic Supplementary Material (ESI) for RSC Advances.
This journal is ©The Royal Society of Chemistry 2016

Supporting Information

Template-free synthesis of salmon pink tube-shaped structure carbon nitride with enhanced visible light photocatalytic activity

Youzhi Cao,[a] Xinbo Jing,[a] Yajuan Chen[a] Wenjie Kang[a] Shufen Wang*[b] and Wei Wang*[b]

[a] School of Chemistry and Chemical Engineering/Key Laboratory for Green Processing of Chemical Engineering of Xinjiang Bingtuan, Shihezi University, Shihezi 832003, China.

[b] College of Sciences,Shihezi University, Shihezi 832003, China.

**wangwei_group@sina.com*

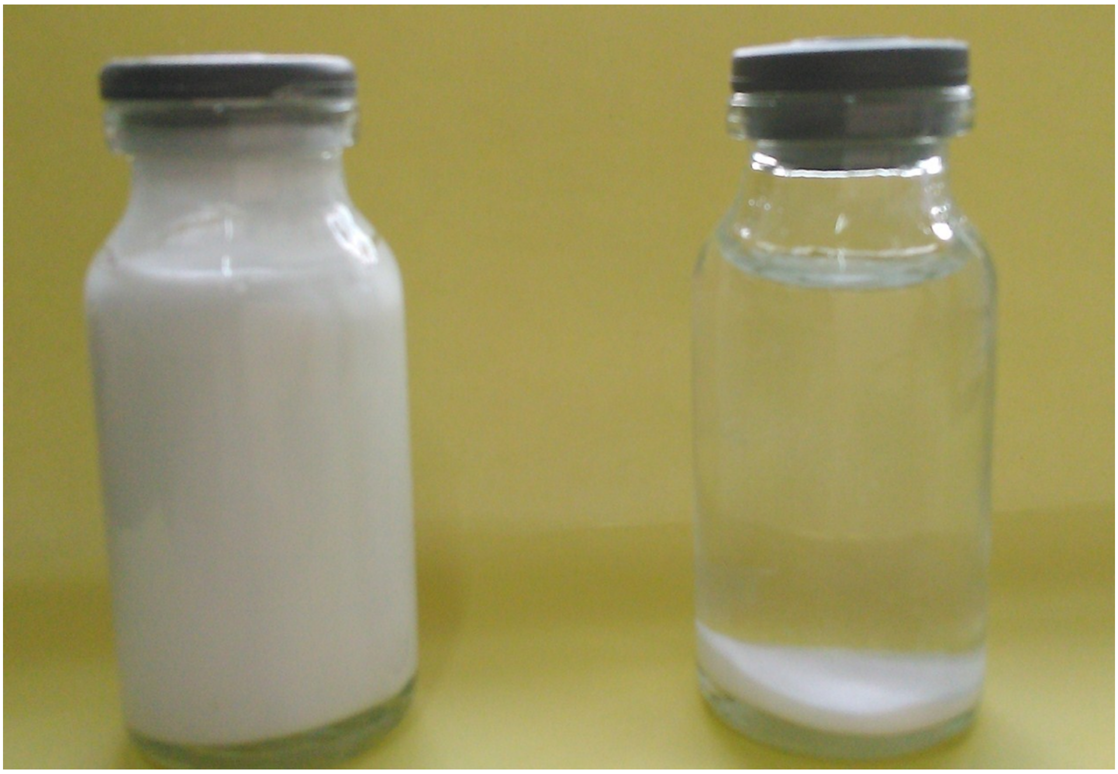

Figure S1 the photographs of reaction phenomena for melamine or M-10-200-24 and 1mol/L HCl.

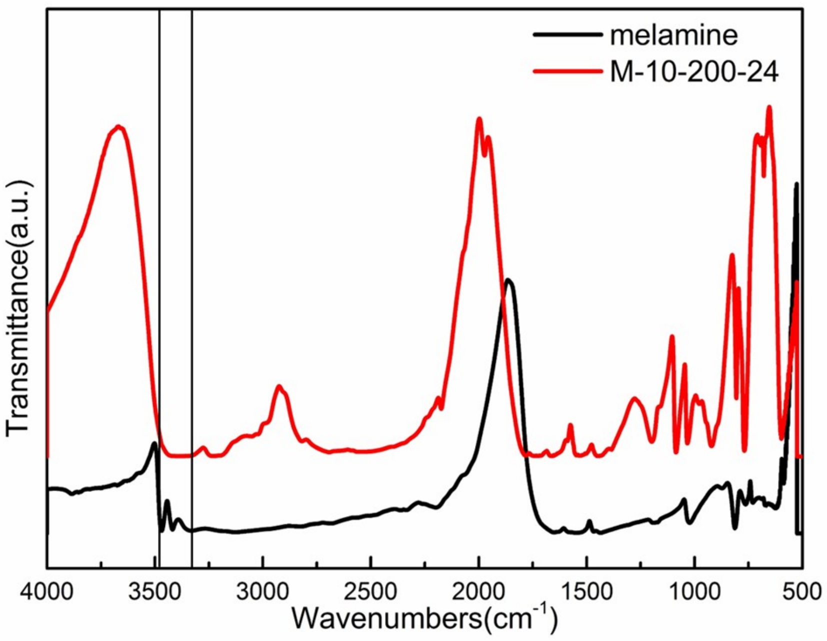

Figure S2. FT-IR spectra of melamine and M-10-200-24.

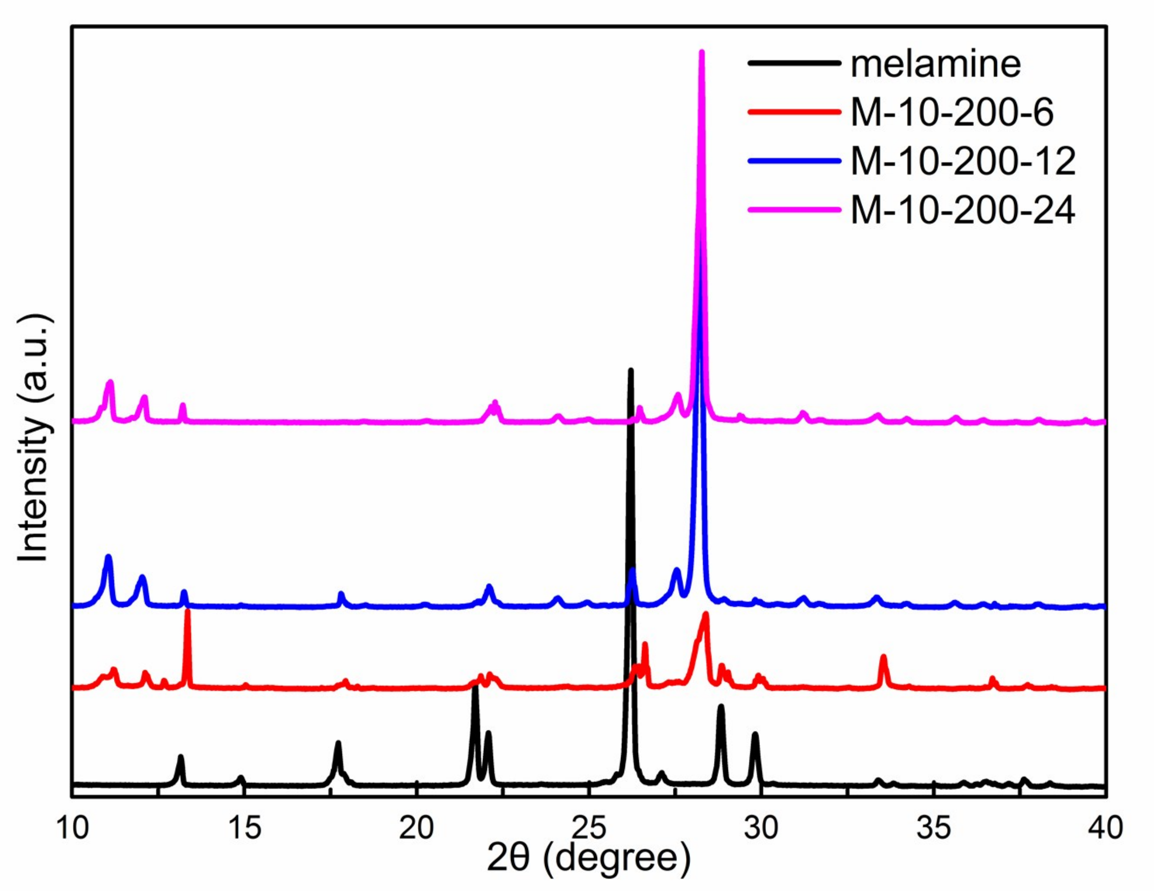

Figure S3. XRDpatterns of monoclinic-phase melamine (raw melamine) and orthorhombic-phase melamine (melamine pretreated with a hydrothermal process).

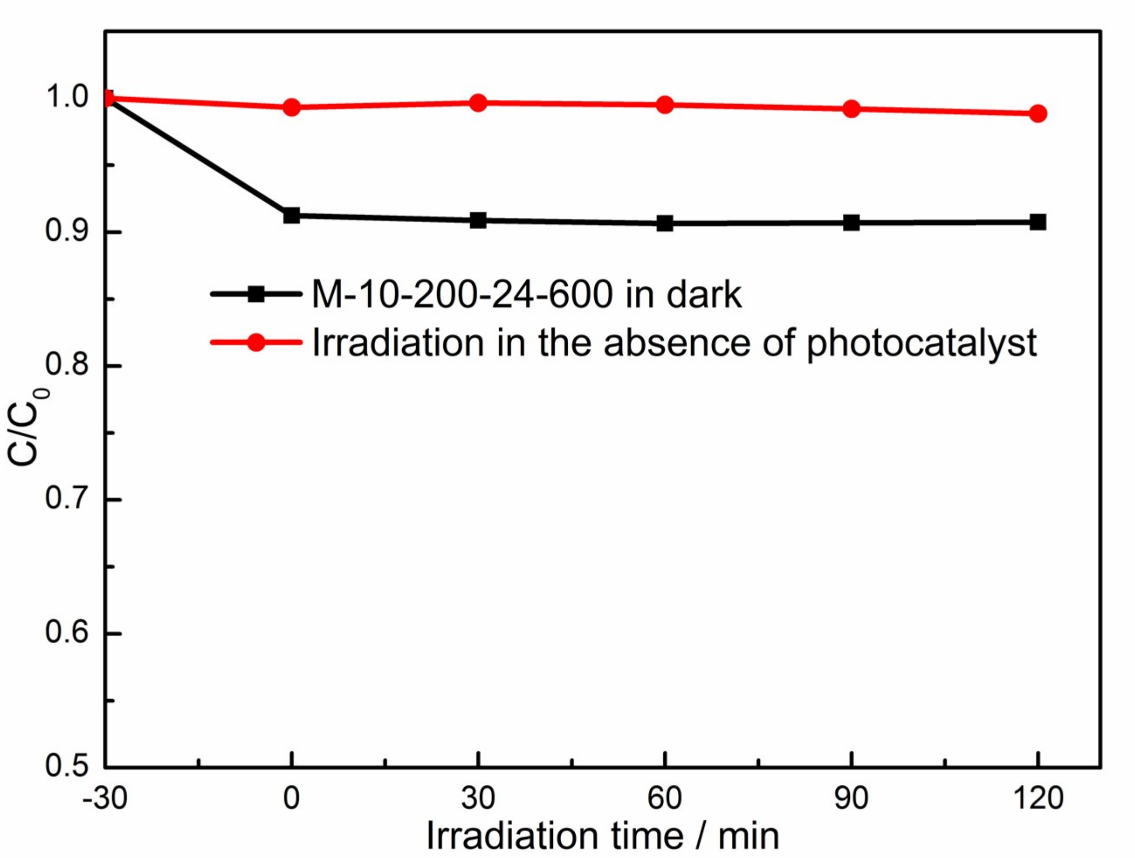

Figure S4 photocatalytic performances for the degradation of MO

Table S1. The deconvolution results of XPS spectra.

	C/N	C1s			N1s			
		C-C	N-C=N		C-N-C	N-[C]$_3$	C-NH2	
		284.94eV	288.3eV	293.85eV	398.75eV	399.8eV	401.1eV	404.5eV
g-C_3N_4	0.7	14%	79%	7%	65%	24%	6%	5%
M-10-200-24-550	0.68	7%	87%	6%	65%	24%	16%	5%
M-10-200-24-600	0.88	29%	66%	5%	68%	19%	6%	7%
M-10-200-24-650	0.67	6%	89%	5%	71%	12%	14%	3%

Table S2. BET specific surface area and pore volume of the samples.

	g-C_3N_4	M-10-200-24-550	M-10-200-24-600	M-10-200-24-650
SBET (m^2/g)	13.7	39.3	60.9	59
Pore volume (m^3/g)	0.07	0.2	0.3	0.26
